# The Malignant Role of Exosomes as Nanocarriers of Rare RNA Species

**DOI:** 10.3390/ijms21165866

**Published:** 2020-08-15

**Authors:** Alina-Andreea Zimta, Olafur Eysteinn Sigurjonsson, Diana Gulei, Ciprian Tomuleasa

**Affiliations:** 1 Research Center for Advanced Medicine-Medfuture, Iuliu Hatieganu University of Medicine and Pharmacy, 400012 Cluj-Napoca, Romania; zimta.alina.andreea@gmail.com (A.-A.Z.); ciprian.tomuleasa@gmail.com (C.T.); 2 The Blood Bank, Landspitali University Hospital, 121 Reykjavik, Iceland; oes@ru.is; 3 School of Science and Engineering, Reykjavik University, 107 Reykjavik, Iceland; 4 Department of Hematology, Oncology Institute Prof. Dr. Ion Chiricuta, 400015 Cluj-Napoca, Romania

**Keywords:** exosomes, snRNA, snoRNA, piRNA, vRNA, yRNA, tRNA, cancer

## Abstract

Nowadays, advancements in the oncology sector regarding diagnosis methods allow us to specifically detect an increased number of cancer patients, some of them in incipient stages. However, one of the main issues consists of the invasive character of most of the diagnosis protocols or complex medical procedures associated with it, that impedes part of the patients to undergo routine checkups. Therefore, in order to increase the number of cancer cases diagnosed in incipient stages, other minimally invasive alternatives must be considered. The current review paper presents the value of rare RNA species isolated from circulatory exosomes as biomarkers of diagnosis, prognosis or even therapeutic intervention. Rare RNAs are most of the time overlooked in current research in favor of the more abundant RNA species like microRNAs. However, their high degree of stability, low variability and, for most of them, conservation across species could shift the interest toward these types of RNAs. Moreover, due to their low abundance, the variation interval in terms of the number of sequences with differential expression between samples from healthy individuals and cancer patients is significantly diminished and probably easier to interpret in a clinical context.

## 1. Introduction 

Cancer represents the second leading cause of death caused by non-communicable diseases. Due to the immense burden that this pathology places on modern society, many of the latest discoveries in molecular and cellular biology have focused on cancer [1]. 

Exosomes (30–150 nm diameter) are membrane-bound vesicles framed within the group of extracellular vesicles (EVs) and are distinguished based on their density, size and origin. These vehicles are loaded with proteins, lipids and a heterogenous profile of ribonucleic acids and are ubiquitously present in most bodily fluids (e.g., plasma, serum, urine, saliva, cerebrospinal fluid and breast milk). Exosomes are able to survive in circulation and even cross anatomical barriers of blood vessels in order to exchange molecular information between different types of secretory and recipient cells, with the final purpose of maintaining the homeostatic balance [2]. In cancer, this mechanism is recapitulated under a pathological context, where exosomes function as active shuttles that influence the behavior of recipient cells in the context of enhanced carcinogenesis, invasion and metastasis, along with drug resistance. In addition to oncogenic cargo, in malignant pathologies, the rate of exosome production and trafficking is significantly increased [3]. Their wide availability in body fluids concomitant with signature cargo for pathological states is promoting the use of these EVs as diagnostic/prognostic biomarkers in oncology and as possible therapeutic targets [4]. 

One of the most studied types of exosome cargo is represented by RNA species, coding and non-coding, that are captured in a controlled and differential manner in these vehicles with further active influence upon the recipient cell. In this context, it was shown that exosomal-shuttle RNAs (esRNAs) are packed into exosomes via RNA binding proteins (RBPs) that form complexes with the RNA species and mediate the transfer during exosome biosynthesis. Exosome analysis showed the presence of 30 RBPs that interact with both exosomal and cellular RNAs; silencing of some of the genes responsible for the translation of RBPs, especially the major vault protein (MVP) transcript, correlates with a reduced presence of esRNAs, showing the functionality of RBP–RNA complexes in exosome shuttling [5]. 

Nowadays, multiple studies on non-coding RNAs are focusing on microRNAs (miRNAs), long-noncoding RNAs (lncRNAs), small-interfering RNAs (siRNAs) and, lately, circular RNAs (circRNAs) [6,7,8]. However, this approach narrows the perspective of the molecular changes in cancer and ignores many other RNA species with higher potential of becoming future biomarkers or therapeutic targets. The term “rare RNAs”, which we use in this manuscript, refers to RNA species of either long or short sequence length that are less frequent in the human genome or that are largely ignored by experts analyzing cancer transcriptomics. Each subtype of rare RNA has specific functions and was proven to be essential in the progression of various cancer types, such as lung cancer [9], head and neck cancer [10,11], breast cancer [12] and colon cancer [13,14] (Table 1). 

The rare RNAs represent a variety of RNA species in terms of biogenesis and mechanisms of action. For our review, we decided to focus on the RNAs that fall into the above-mentioned category and that are found in exosomes. These are yRNAs, transfer RNA (tRNA) fragments (tRF), tRNA halves (tiRNA), small nucleolar RNA (snoRNA), small nuclear RNA (snRNA), vault RNA (vRNA) and piwi-interacting RNA (piRNA) (Table 1). These RNAs are increasingly correlated with cancer exosomal loading, they are generally highly conserved across species, and they participate in essential biological processes. 

yRNAs consist of a group of four RNAs that are transcribed, in humans, from specific yDNA regions, named hY1, hY3, hY4 and hY5. As single units, yRNAs are involved in DNA replication. When bound to the Ro60 protein, they participate in maintaining RNA stability [15]. yRNAs can be fragmented into smaller particles, named small yRNAs (s-RNYs), that have the function of translation repression [16]. 

tRNA is a well-known non-coding RNA with an essential role in translation; however, it was later discovered that this RNA species can be fragmented into tiRNAs and tRFs that regulate gene expression at the epigenetic level and participate in translation repression [17]. 

snRNAs are encoded by special regions of the DNA called U1, U2, U4, U5 and U6 that also give their name. snRNAs have low variability and are an essential part of the spliceosome machinery during processing of primary messenger RNA (mRNA) [18]. snoRNAs are encoded in the introns and intergenic regions of the DNA. snoRNAs are also part of the spliceosome machinery. In addition to this, snoRNAs can also participate in the 2′-O-methylation or pseudouridylation of ribosomal RNA (rRNA) [19,20]. 

vRNAs are found in the cytoplasm in ribonucleoprotein complexes called vaults. These are highly conserved sequences across species and have been known for many years to be involved in viral replication and cytoplasmic/plasma membrane trafficking of biomolecules. In cancer, vRNAs are involved specifically in autophagy and in the extracellular export of cytotoxic therapeutics during the process of acquired drug resistance [21,22]. 

Alu-containing RNAs (Alu-RNAs) are transcribed from transposable element, containing Alu repeats that offer new promoter/enhancer sites for transcription. Alu-RNAs have also been found to participate in the modulation of miRNA expression [23,24,25,26]. 

piRNAs have been initially discovered in the male gonadal cells, with the primary role of protecting germinal cells from transposable elements, especially those of viral origin [27]. piRNAs are specifically bound to piwi proteins and form the ping pong pathway. In malignant cells, they participate in the epigenetic regulation of DNA [28] and are essential for maintaining cancer stemness [29]. 

The pseudogenes are formed from protein-coding genes that have acquired new loss-of-function mutations. These DNA areas were for a long time believed to be transcriptionally inactive; however, the transcripts of these pseudogenes are now known to form endogenous siRNAs or act as decoys for miRNA-mediated repression of translation, especially miRNA interaction with their gene-of-origin transcripts [30].

In the present manuscript, we decided to focus on the role of rare RNA species as biomarkers of various cancers, due to their presence in the circulating exosomes and differential expression between healthy controls and cancer patients. 

## 2. Exosomal Loading of Rare RNAs in Cancer 

### 2.1. Exosomal Loading of Rare RNAs in Breast Cancer and Their Roles in Development and Progression 

Breast cancer is the second most common type of cancer in women and the leading cause of cancer-related death in women [31]. This type of cancer has both genetic [32] and lifestyle causes [33].

In the case of RNASeq analysis of MCF-7 (human double positive breast cancer) and MCF-10A (human mammary epithelial cells) cell lines, miRNAs were the most common RNA species, comprising 43% of the total analyzed small reads from the intracellular environment and approximately 11% of the total RNA species in the intracellular environment. In contrast to this, in exosomes released by these two cell lines, miRNAs represent less than 1% of the analyzed transcripts. In exosomes, rRNA and tRNA fragments are the dominant components of the mapped reads, constituting approximately 10% and 62%, respectively. yRNAs have a uniform distribution in the two compartments: intracellular and extracellular vesicles. vRNAs are present in very low levels in the intracellular space and only have modest representation in the exosomes; the difference was not significant in this case [34]. RNA profiles of MCF-7 and MCF-10 cell lines identified over 100 piRNAs, with some of them being differentially expressed between the breast cancer cell line and the immortalized breast epithelial cell line, MCF-10A [35]. The existence of these RNA species within the intercellular space is raising the possibility of piRNA transfer in exosomes derived from breast cancer cells and dynamic differential expression between healthy and malignant tissue [35,36]; however, further investigations are necessary to confirm this hypothesis. tRNA fragments (tiRNAs and tRFs) can form dimers or G-quadruplex structures that confer on them more stability during exosomal transport. In a study by Gámbaro F. et al., the double positive breast cancer cell line MCF-7 was transfected with 5′ tiRNAGly. This tiRNA was later found to be exported through exosomes and successfully received in the recipient cells. The intracellular level of 5′ tiRNAGly was positively correlated with the exosomal level in a dose-dependent manner, thus showing specificity for the exosomal loading of this non-coding RNA [37].

### 2.2. Exosomal Loading of Rare RNAs in Lung Tumors and Their Roles in Development and Progression

Lung cancer represents the cancer with the highest mortality rate, affecting especially the male population, with more than 1.7 million deaths worldwide [31]. This disease is very hard to diagnose in incipient stages, even in the context of modern day advances in clinical diagnostics. Even a small tumor can rapidly migrate and metastasize to novel sites within the body [38]. Therefore, there is an urgent need to discover reliable biomarkers that are detectable through minimally invasive procedures. 

Savelyeva A.V et al., through differential centrifugation of blood samples from patients, revealed an array of biomarkers. In the 160,000× *g* fraction pellet enriched with EVs isolated from the blood of non-small cell lung cancer (NSCLC) patients and healthy donors, in NSCLC, the following snoRNAs were upregulated: SNORD113 and SNORD78. Meanwhile, the following snoRNAs were downregulated: SNORA33 and SNORD1B. In the supernatant of the 160,000× g fraction of NSCLC samples, SNORD113 was again upregulated and SNORD50D, SNORD18A, SNORD59B, SNORD48, SNORD81 and SNORD29 were downregulated [39]. snRNAs U1, U5 and U6 were also enriched in the 160,000× g fraction containing circulating exosomes from NSCLC patients [39]. 

### 2.3. Exosomal Loading of Rare RNAs in Oral Tumors and Their Roles in Development and Progression

Oral cancer comprises the malignant transformation of epithelial cells from the oral cavity. The main risk factors for this disease are betel quid chewing, alcohol consumption, smoking tobacco and human papilloma virus (HPV) infection [40,41]. 

Human whole saliva (WS) is abundant in clinically discriminatory RNA species that can become useful in the minimally invasive diagnosis of oral cancers; these RNA species are found in free form, inside cancer circulating cells or loaded into exosomes [42]. RNA sequencing and genome mapping derived from WS exosomes versus WS showed a high abundance of miRNA species but also highlighted the presence of piRNAs and snoRNAs. Interestingly, exosomes were associated with a significant abundance of piRNAs in comparison with the other biological samples. piR-39980 is upregulated in exosomes, while piR-61648 is overexpressed in WS. In the WS, two types of exosome (exosome 1 and exosome 2) with different sizes and proteome profiles were identified. piRNAs and snoRNAs also have specific profiles between these two types of exosome. piR-39980, piR-48209, piR-52207, piR-38581 and piR-36095 and U78, U44, U21, U31 and U104 are the most abundant piRNAs and snoRNAs from exosome 1. piR-39980, piR-59293, piR-61648, piR-55361 and piR-41405 and U78, U21, U44, U31 and U27 have the highest levels in exosome 2 [42]. 

These initial observations demonstrate that WS is abundant in exosomes that contain rare RNA species, a fact that can be extrapolated to possible studies regarding the minimally invasive diagnosis of oral malignancies through assessment of differential types of exosomal rare RNA cargo. A later study showed that piRNAs are surprisingly predominant in human cell-free saliva (CFS) in comparison with other fluid samples or intercellular ones, while the level of miRNAs was found to be similar between CFS, serum and cerebrospinal fluid [43]. 

### 2.4. Exosomal Loading of Rare RNAs in Gastrointestinal Tumors and Their Roles in Development and Progression

Gastrointestinal tumors comprise 26% of new cases globally and 35% of cancer-related deaths worldwide. This group of malignancies places a major burden on public health and, often, cases are preventable through lifestyle changes [44]. 

In hepatocellular carcinoma (HCC), miRNAs and yRNAs are the most abundant RNA types in plasma exosomes; however, the most differentially expressed RNA species in HCC patients versus healthy controls were lncRNAs and snoRNAs. SNORD3A, SNORD91B, SNORD65 and SNORD55 were upregulated in HCC, while SNORD116-3 and SNORD116-24 were downregulated [45]. The signal recognition particle RNA (srpRNA) is the RNA component of the signal recognition particle, a ribonucleoprotein (RNP) that control cotranslational protein targeting, through which proteins are send to the endoplasmic reticulum for processing [46]. srpRNA RN7SL1S was identified as the most upregulated RNA species in the extracellular vesicles from HCC patients. The overexpression of this srpRNA enhances the proliferation and colony formation capacity of HCC cells in vitro [45]. In the culture media from the SK-Hep1 liver cancer cell line, in the exosomes, the predominant non-coding RNA type is rRNA (30%), followed by miRNA (22%), snRNA (9%), tRNA (5%) and snoRNA with less than 1%. tRNA fragments show differential expression between liver cancer patients, with 35 upregulated and 11 downregulated transcripts. Liver cancer patients have higher expression of tRNA-ValTAC-3, tRNA-GlyTCC-5, tRNA-ValAAC-5 and tRNA-GluCTC-5, in comparison with exosomes from plasma of healthy donors [47]. 

Five snoRNAs (SNORA14B, SNORA18, SNORA25, SNORA74A and SNORD22), present in the culture media from pancreatic cell lines, were encapsulated in the exosomes. During an observational clinical trial comparing the expression of circulating encapsulated RNAs between 27 pancreatic cancer patients and healthy controls, it was discovered that SNORA74A and SNORA25 have greater power to distinguish between the two groups compared with the commonly used biomarker, CA19-9, thus rendering these two snoRNAs potential biomarkers of pancreatic cancer [48]. piRNAs and tRNAs have a low level of representation in plasma from pancreatic cancer patients. However, they have a higher potential of becoming disease biomarkers that could be more specific than miRNAs. tRNAs and piRNAs from plasma exosomes can differentiate between healthy individuals and pancreatic cancer patients and between different types of pancreatic cancer, such as intraductal papillary mucinous neoplasm (IPMN) and pancreatic ductal adenocarcinoma (PDAC). The following tRNAs are overexpressed in pancreatic cancer versus healthy individuals: tRNA125-Thr CGT, tRNA21- Ser TGA, tRNA15-Cys GCA, tRNA55-Ile-TAT, tRNA5-ILE TAT. With a fold change (FC) above 15, in pancreatic cancer versus healthy individuals, tRNA125-Thr CGT can be a biomarker for this disease. At the same time, tRNA21-Ser TGA can discriminate between the two pancreatic cancer types (PDAC and IPMN). It is significantly overexpressed in PDAC versus IPMN (FC = 6.73). The same study also found that the following piRNAs are upregulated in pancreatic cancer: hsa-piR-52959, hsa-piR-53108, hsa-piR-30690, hsa-piR-54479, hsa-piR-56621, hsa-piR-54888, hsa-piR-42185, hsa-piR-46410, hsa-piR-58897, hsa-piR-58897. The overexpression of these piRNAs is more specific for PDAC than IPMN [49].

In a study comparing the exosomal RNA content from plasma of gastric cancer patients versus healthy controls, it was proven that tRF-25, tRF-38 and tRF-18 have significantly higher expression in gastric cancer; it follows that these RNAs can accurately predict the diagnosis of this malignancy [50]. The exosomes from four gastric cancer cell lines, MKN45, SGC7901, NCI-N87 and AGS, compared to the ones obtained from a normal cell line, GES-1, were analyzed through deep sequencing in order to classify the abundance of RNA species found in extracellular vesicles. As expected, miRNAs accounted for approximately 25% of the mappable counts, followed by rRNA, tRNA, snRNA, snoRNA, piRNA and yRNA [51]. In the context of a diagnosis proposal, the limited range of variation of specific rare RNA, implication in various molecular processes and high stability (due to secondary structures or by being part of RNPs) in circulation favor the use of these RNAs as minimally invasive disease biomarkers. For instance, the use of piRNAs (piR_651 overexpression) [52] or tiRNAs (tiRNA-5034-GluTTC-2 downregulation) [53] as circulating gastric cancer biomarkers can be proposed.

Even the same cell line of colon cancer can produce different kinds of extracellular vesicles (EVs) in terms of RNA profile. LIM1863 cell line releases two types of exosome: from the apical part (Ep-CAM-Exo) and from the basolateral part (A33-Exo). The pseudogene transcripts are especially located in the A33-Exo. Of these, the most abundant are RPL41P1, RPL39P3, EEF1A1P5, CTD-2031P19.4, CTB-63M22.1, RP11-742N3.1, RP11-122C9.1, RPL9P8, RPL9P9 and RP11-466H18.1. In these exosomes, a type of RNA with a yRNA-like sequence was enriched; however, its exact function is still unknown. snRNA U6 has a significantly higher level in EpCAM-Exos compared with A33-Exos. In regard to snoRNAs, SNORA18, SNORA27, SNORA57, SNORA62, SNORA68, SNORA70, SNORA77, SNORD23 and snoU13 are overexpressed in the A33-Exos, while SNORA14B, SNORA31, SNORA76, SNORA77, U3 and snoU13 are enriched in the Ep-CAM-Exos [54]. 

### 2.5. Exosomal Loading of Rare RNAs in Billary Tract Tumors and Their Roles in Development and Progression

Gall bladder carcinoma (GBC) and cholangiocarcinoma (CCA), both cancers of the biliary tract, are associated with poor prognosis and a high recurrence rate. In this sense, the identification of minimally invasive diagnosis strategies by means of circulatory biomarkers able to discriminate incipient stages becomes especially important. RNA sequencing of exosomes from plasma of GBC (*n* = 4) and CCA (*n* = 5) patients compared to healthy ones shows 514 and 730, respectively, altered piRNAs, with most of the sequences found to be overexpressed. Some of the rare RNAs were upregulated in both malignancies, demonstrating a general role of these piRNAs in carcinogenesis or specificity for biliary tract cancers. Importantly, piR-10506469 was further validated in blood samples from a large cohort of patients with GBC and CCA as being significantly increased compared to healthy controls. The same analysis of patients that underwent resection therapy showed that this specific piRNA decreases after tumor removal, together with piR-20548188 [55]. 

### 2.6. Exosomal Loading of Rare RNAs in Brain Tumors and Their Roles in Development and Progression

The most common brain tumors originate in glial cells and form gliomas, among which the most aggressive forms are represented by glioblastomas. The tumors do not invade new sites of the body but are highly aggressive due to their location and the rapid degradation of brain function, with associated accelerated decrease in the quality of life [56,57]. 

There is a difference in the non-coding RNAs’ cargo from microvesicles and parent glioma cell lines (U251) that alters the behavior of recipient cells (vascular endothelial cells). Besides miRNAs, which represent more than 90% of exosome cargo, there are vRNAs and antisense intron-derived RNAs. EVs are enriched in the following vRNAs: VTRNA1-1 (the most abundant), VTRNA2-1, VTRNA1-2, VTRNA1-3, VTRNA3-1p [58]. 

From the total levels of RNA cargo from glioma cells, piRNAs represent the second most frequent RNA species, followed by snRNAs, snoRNAs and rRNAs. From these, the following piRNAs were enriched in EVs from glioma cell lines: hsa_piR_019675, hsa-piR_020388, hsa_piR-020829, hsa_piR_020381, has_piR_016735, has_piR_020365, ha_piR_004153, hsa-piR_019825, has_piR_015249, has_piR_009059, has_piR_000753, has_piR_008488. SNORD20 occupies the eighth place among the most expressed RNAs in glioblastoma cell lines [59]. When comparing the RNA content of exosomes in four glioma cell lines, it was proven that their exosomes and extracellular ribonucleoproteins have different RNA species patterns. yRNAs are among the dominant RNA types (along with snRNA and repeat RNA) in exosomes isolated from GBM4 (grade 4 glioblastoma); however, they were almost absent in the exosomes from GBM8 (glioblastoma stem cells) and had overall low expression compared to the mean expression of all exo-glioma cell lines. Upon analysis of RNPs versus intracellular components, yRNAs were significantly overexpressed in the RNPs versus intracellular components and showed uniform distribution throughout all cell lines. snRNA had uniform overrepresentation in all glioma cell exosomes, except for MGG75 (intracranial glioma), which showed slight underrepresentation. tRNAs were also very underrepresented in glioma exosomes, whereas in RNPs, they were the most abundant RNA species [60]. 

Glioblastoma cells secrete, in vitro and under hypoxic conditions, exosomes that contain protein-lysine 6-oxidase (LOX), thrombospondin-1 (TSP1), vascular endothelial growth factor (VEGF) and metalloproteinase with thrombospondin motifs 1 (ADAMTS1). Upon delivery in targeted glioblastoma cells, these exosomes induce upregulation of SNORD1 (SNORD116-21), along with other lncRNAs. This causes increased viability of glioblastoma cells and, in endothelial cells receiving these hypoxic exosomes, causes increased ability of tube formation, thus showing the malignant role of exosomes containing SNORD1 [61]. yRNAs are many times enriched in exosomes because they are involved in the degradation of misfolded RNAs. SnoRNAs are overall more common in cells than in exosomes; however, some snoRNAs were found to be more abundant in exosomes isolated from endothelial cells than in the cells of origin. These are SNORD78, SNORD93, SNORD114-22, SNORD43, SNORD114-9, SNORD104, SNORD119, SNORD114-24, SNORD100, SNORD82, SNORD12, SNORD99, SNORD69, SNORD66, SNORD12B [62]. vRNAs and yRNAs are the most common types of RNA in the exosomes of endothelial cells, besides miRNAs. VRNA1-3 and VRNA2-1 are abundant in exosomes and the 5′ fragment of RNY5 [62]. 

Medulloblastoma is a very aggressive type of brain tumor that originates in neuronal stem cells. Medulloblastoma cells secrete exosomes in larger quantities than normal fibroblasts. In these exosomes, transposable RNA/DNA elements, besides DNA fragments, were found. The retrotransposon elements from HERV, Alu and L1 repeats had higher levels in exosomes than in the cells of origin [63]. 

### 2.7. Exosomal Loading of Rare RNAs in Melanoma and Their Roles in Development and Progression

Melanoma develops from the malignization of melanocytes and is the most aggressive type of skin cancer, with rapid progression toward metastatic phases and reduced survival rate. Half of the metastatic patients have the BRAF somatic missense mutation, especially at the amino acid residue V600 (BRAFV600) [64]. Development of BRAF inhibitors vemurafenib and dabrafenib has significantly increased the survival rate of melanoma patients; however, the installation of drug resistance often occurs. To assess the dynamic changes upon vemurafenib treatment, researchers analyzed the RNA and protein content in EVs, including exosomes, after vemurafenib administration in melanoma cell lines and patient-derived xenografts (PDX) [65]. Exosomes isolated from the cell media of treated cells showed increased concentrations of total RNA and also misbalanced RNA/protein ratios (the RNA content was also found to be increased in other EVs, namely microvesicles and apoptotic bodies, but the RNA/protein ratio was not affected). Small RNA deep sequencing of exosomes, microvesicles and apoptotic bodies showed the presence of yRNA, snRNA, tRNA, snoRNA, rRNA, lincRNA, piRNA, miRNA and mRNA. The small RNA profile from exosomes was characterized by an increased concentration of miRNAs compared with the other two EVs and a unique set of snoRNAs distinct from microvesicles, apoptotic bodies and cells. Moreover, tRNAs were found to be mainly associated with EV content, with valine tRNA increased in exosomes compared to cells and the other two types of analyzed EVs. Finally, although the proportion of the ncRNA species did not significantly change upon vemurafenib administration, the expression of individual ncRNAs was altered, e.g., miR-211–5p upregulation in EVs [65]. 

### 2.8. Exosomal Loading of Rare RNAs in Bone Cancer and Their Roles in Development and Progression

Osteosarcoma is the most common and aggressive forms of bone cancer. It develops from osteoblasts found in bones. One of the major challenges that this disease poses is frequent recurrence and metastasis formation [66]. A theory was recently formed that, in p53 mutated (p53R172H) osteosarcoma cells, the Ets2 transcription factor stimulates the transcription of over 24 snoRNAs, which leads to further mutations in the tumor cells or, through exosomal transfer, they may play a significant role in metastatic niche formation in lung tissue [67]. Ewing sarcoma is a type of rare bone or soft tissue cancer that originates in mesenchymal stem cells. It was proven that the cancer cells of Ewing sarcoma secrete exosomes loaded with retroelement RNA, which sustains inflammation and migration [68]. 

### 2.9. Exosomal Loading of Rare RNAs in Hematological Malignancies and Their Roles in Development and Progression

Hematological malignancies comprise a group of cancers that arise from the different progenitor cells of hematopoiesis in the bone marrow and lymphoid organs [69,70]. The malignancies that originate in the lymphoid lineage are represented by acute lymphoblastic leukemia (ALL), chronic lymphoblastic leukemia (CLL), lymphoma and multiple myeloma (MM). The hematological cancers that arise from myelogenous lineage are named myelodysplastic syndromes (MS), acute myelogenous leukemia (AML), chronic myelogenous leukemia (CML) and myeloproliferative neoplasms. As opposed to solid tumors, these groups of malignancies present high heterogeneity in terms of development and treatment approaches [71]. 

yRNAs are the predominant small RNA species, besides miRNAs, found in exosomes isolated from pediatric patients diagnosed with anaplastic large cell lymphoma (ALCL). A comparison was made between the plasma of pediatric patients with NPM-ALK fusion gene and the plasma from healthy donors. Five ALCL cell lines were also taken into consideration. Moreover, in relapsed patients, the levels of full length RNY4 are significantly more abundant than in the case of ALCL patients in complete remission [72]. 

Analysis of exosomes isolated from bone marrow supernatant of MM patients highlighted the abundant presence of another rare RNA species: piR-004800 (piRNA exosomal content represents approximately 20–30% of that of the total exosomal RNA). The same piRNA was found to be upregulated also in primary MM cells. Functional studies involving the experimental inhibition of the piRNA reduced cell proliferation rate both in in vitro and in vivo models, accompanied by increased apoptosis rate. The pathological mechanism was positively associated with the sphingosine-1-phosphate receptor (S1PR) signaling pathway, where S1PR is concomitantly expressed and functionally interconnected with piR-004800, with further positive regulation of the PI3K/Akt/mTOR signaling pathway [73]. 

Stem cell isolation and manipulation is currently being explored in immunotherapy after allogeneic stem cell transplantation in hematological malignancies. Importantly, researchers are investigating the most suitable stem cells for specific applications by analyzing their molecular content and phenotypical behavior. Bone marrow mesenchymal stem cells (BMMSCs) and stem cells from the apical papilla (SCAP) of teeth were compared in terms of their piRNAs exosomal cargo content in order to highlight the potential signaling pathways modulated by the secreted vesicles [74]. Previous studies have shown that the exosomes secreted by MSCs exhibit synonymous functions with the cell itself in terms of regeneration, inhibition of inflammation, immunoregulation and tissue repair [75,76,77]. piRNA expression profiling revealed 593 and 920 piRNAs, with a median length of 21 nucleotides derived from exosomes secreted by SCAP and BMMSCs, respectively. hsa-piR-020326, hsa-piR-016735 and hsa-piR-017716 were identified as being highly expressed in SCAP, while hsa-piR-016735 demonstrated high expression in BMMSCs; moreover, 21 miRNAs were identified as being differentially expressed between the exosomes secreted by the two types of cells. Gene enrichment analysis associated the differentially expressed piRNAs with metabolic processes, biological regulation, binding and catalytic activity and cellular processes, all pathways being previously connected with the function of MSCs [74]. The RNA deep sequencing of plasma EVs revealed that indeed the miRNAs are the most abundant RNA species loaded into these vesicles: they constituted 76.20% of mappable reads, followed by piRNA (1.31%), tRNA (1.24%), snRNA (0.18%) and snoRNA (0.01%) [78]. However, these profiles are highly dependent on the source cell. For instance, in the exosomes from mesenchymal stem cells (MSCs), miRNAs represent only 2–5% of loaded RNAs. Other RNA species have significant fold change differences between MSCs and their secreted exosomes: pseudogenes (3.83 FC), rRNA (3.13 FC), tRNA (2.46 FC), snRNA (−3.41 FC), snoRNA (−6.65 FC). tRNA halves represent the dominant parts of exosomes from MSCs from adipose tissue, while miscRNA is the dominant component in bone marrow MSCs; it follows that this profile is dependent on the cell of origin [79]. MiscRNA is the term used for miscellaneous RNA, referring to small RNA sequences that function as enzymes and translation repressors and induce RNA degradation [80].

circRNAs were also considered one of the rare RNA species until recently, when the interest in these RNA species was greatly enhanced. As a result of this, the focus of this review was not circRNAs; however, there is a type of circRNA that is still widely ignored. The majority of circRNAs studied are of nuclear origin, meaning that they are encoded by the nuclear genome. There is, however, a type of circRNA that is encoded by the mitochondrial genome, namely the mitochondrial circRNA (mcRNA). In chronic lymphocytic leukemia (CLL), the mcRNA, mcCOX2, left after the processing of the COX2 gene, was found to be overexpressed in plasma exosomes from CLL patients compared with normal controls. During in vitro simulation, the CLL cells overexpressing mcCOX2 have enhanced proliferation and apoptosis resistance ability; thus, mcCOX2 stimulates leukemogenesis [81].

## 3. The Clinical Impact for New Biomarker and Therapeutic Target Discovery—Importance of Deep Sequencing and Data Mining

**Figure 1:**
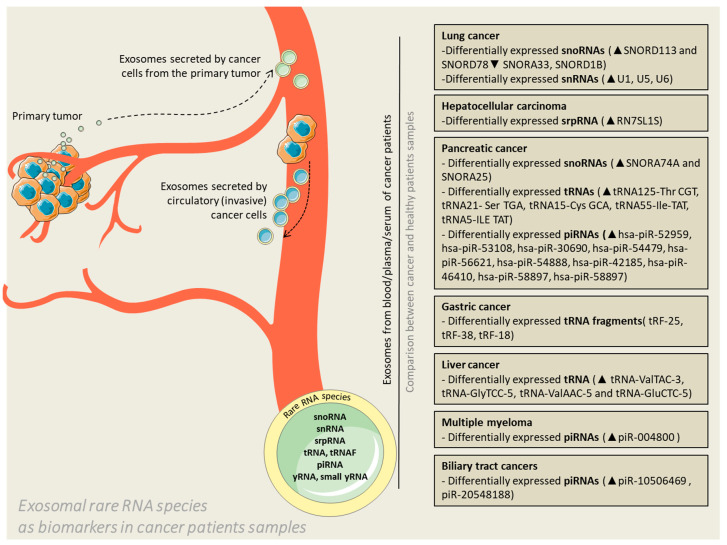
Exosomal rare RNA species as biomarkers in cancer patients’ samples. Exosomes are released by cancer cells in the extracellular environment with the purpose of facilitating communication with neighboring or distant cells. The vesicles can cross the blood barrier and enter the circulation; therefore, liquid biopsies conducted by means of blood, plasma or serum samples can become reliable diagnostic samples or even predictors of potential therapeutic targets. The context of exosomes (represented in the figure by rare RNAs) is the actual substrate for differentiation of patients in terms of health status.

All of the studies presented in this review, which discovered differential patterns of RNA species loading in exosomes versus donor cells or exosomes from patients versus healthy controls (Figure 1), have in common the use of next generation sequencing, especially deep sequencing, of the RNA and data mining of publicly available databases.

**Figure 2:**
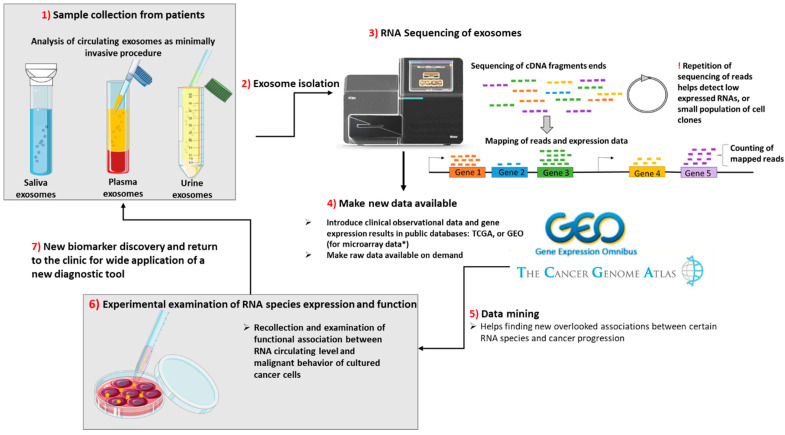
From the collection of samples from patients undergoing minimally invasive procedures until the discovery of new biomarkers of a cancer type or for the assessment of cancer progression, there are 7 steps to be taken. (**1**) The samples are collected from patients and processed separately by centrifugation at low speed for the separation of exosomes from other heavier particles, such as cells. Then, through high speed ultracentrifugation or differential centrifugation, the next step is taken. (**2**) Exosome isolation. (**3**) The purified exosomes are sequenced during RNASeq: first, the total or small RNA is purified; then, it is reversed transcribed into cDNA, fragmented and labeled. The cDNA fragments are sequenced, and the reads are mapped to the genome of reference. During deep sequencing technologies, the repetition of sequencing is greater; thus, the genes that have a very low level of expression can be more easily identified. (**4**) The RNA Seq or microarray data (the microarray technology was not presented) should be made public by uploading the sequencing data along with clinical characteristics of the analyzed samples on publicly available databases, such as the Cancer Genome Atlas (TCGA) or Gene Expression Ominibus (GEO). In addition, if required, the authors should provide the RNASeq raw data. (**5**) Data mining. By accessing the above-mentioned databases, other scientific groups may find new associations between gene expression and clinical data. (**6**) In order to test for a causative correlation between RNA species expression and function, the in vitro testing and collection of exosomes from supernatant are needed. After definitive results in pre-clinical data, if the new RNA species show high sensitivity and specificity, comparable with that of the current biomarkers, they will be introduced in common clinical practice. (**7**) New biomarker discovery for wide application of a new diagnostic tool.

The sequence of events from sample collection to discovery of new biomarkers based on “rare” RNA species from exosomes is presented in Figure 2. 

The technology of next generation sequencing has rapidly evolved in the last few years, concomitant with decreased costs and time spent on the procedure. One direction is the increase in sequencing depth and read length. Sequencing depth of coverage is the read length multiplied by the number of reads and divided by the haploid genome length [82]. The most important aspect of increasing the sequencing number of reads is that less frequent mutations from a small population of cells in a sample or low represented cell clones are detected [83]. Single cell RNASeq also offers this great advantage of detecting unique transcriptional profiles and thus new RNA species that also may follow linage development and new correlations between genes [84]. However, for rare RNAs to be discovered in exosomes, the option of deep RNASeq is the “gold standard”. Moreover, the increase in read length can help scientists to find new *cis-* or *trans-* co-expression of genes, discovering longer transcriptionally active sites of the DNA and sites of the DNA that are enriched in tandem repeats [83,85]. The advantage of using advanced deep sequencing technologies that are able to process longer reads is that we can discover new lncRNAs with unknown functions and roles in disease progression that will deepen our current understanding of the molecular mechanisms of cancer development and progression, while creating new therapeutic targets with future potential for drug discovery or repositioning. 

However, large quantities of data are difficult to process; thus, more and more advanced bioinformatic tools that can keep up with this information will also be needed. The first step toward understanding the newly discovered lncRNAs is to look at the RNAs that are the origin of short non-coding RNAs and whose functions are easier to determine [86]. The sequencing technology can provide valuable information, but it will be difficult to assess a single type of molecular interaction, considering that cancer is a polyfactorial disease. Moreover, what is known so far is that these sequences have very low levels of expression; thus, it is easy to assume that their function is not fundamental for cancer cell survival. In addition to these, there is the financial aspect; if all this technological development and financial investment in discovering the role of rare RNAs will lead to a dead end, it will not be profitable to obtain funding for this area of research. To overcome this challenge, the second approach to “rare RNA” function discovery, namely data mining, is important.

Data mining from public datasets, such as the Cancer Genome Atlas (TCGA) or Gene expression omnibus (GEO), is essential for understanding the role of these rare RNAs and their specificity toward certain types of cancer. For instance, the pan-cancer analysis of TCGA RNASeq data reveled a significant implication of snRNAs especially in cancers of the digestive tract. For instance, RNU6-101 P is a risk factor for esophageal cancer, while RNVU1-4 is a protective factor for stomach cancer [87]. In prostate cancer, with the help of data mining from GEO, by means of re-analyzing raw data and applying differential expression analysis between seminal fluid from controls and prostate cancer patients, it was proven that 5′ tRNA halves have increased expression levels in seminal fluid from prostate cancer patients, thus creating a new path for non-invasive biomarker discovery [88].

## 4. Final Remarks and Conclusions 

The cancer-driven role of rare RNA loading in exosomes is becoming increasingly recognized with the development of databases containing the specific expression of these RNAs (http://bioinformatics.zju.edu.cn/OncotRF/index.html), such as OncotRF, a database based on TCGA analysis of various cancer samples that can show the expression of tRFs in various cancers [89]. The “rare” RNA species are often among the dominant RNAs in exosomes and some of them show clear specificity to a cancer type. Due to the high degree of stability of these RNAs, low variability and, in most cases, conservation across species, rare RNAs can be considered a more reliable source of biomarkers for non-invasive investigation of cancer than commonly used miRNAs or lncRNAs. 

**Table 1:** Characteristics of rare RNA species included in the present narrative review.

Type of RNA	Sequence Length (nt)	Region of the DNA	Type of Polymerase	Degree of Conservation across Species	Function	Biological Role	Type of Cancer	Cellular Localization	Ref.
yRNA	84, 98, 101, 112	From specific DNA regions named hY1, hY3, hY4 and hY5; there are also pseudogenes of these YDNA regions	RNA Pol III	High degree of conservation	DNA replication; RNA stabilization (when bound to Ro60 protein)	Cell proliferation; cell cycle progression	Lung cancer, prostate cancer, colon cancer, renal cancer, cervical cancer, bladder cancer	Nucleus, cytoplasm	[90,91,92]
small yRNA	19–60	Represent fragments of yRNAs, especially from 5′ end of yRNA	RNA Pol III	High degree of conservation	Translation repression	Cell death, inflammation	Oral cancer, breast cancer	Cytoplasm	[11,16,93,94,95,96]
tRF	14–30	It has 446 specific genes in humans (for the full-length tRNAs)	RNA Pol III	Full length transcripts have high degree of conservation	Epigenetic control through heterochromatin modulation; competes with Dicer for miRNA binding; translation repression of mRNA; involved in RNA degradation and control of RNA stability	Cell cycle progression, cell proliferation, change in metabolic pathways, metastasis formation	Bladder cancer, lung cancer, renal cancer, colon cancer, breast cancer, ovarian cancer, liver cancer, lymphoma, chronic lymphocytic leukemia	Cytoplasm	[97,98,99,100,101,102]
tiRNA	31–40	446 specific genes in humans (for the full-length tRNAs)	RNA Pol III	Full length transcripts have high degree of conservation	Chromatin/epigenetic modifications, translation repression, miRNA-like function; involved in RNA degradation and control of RNA stability	Cell cycle progression, cell proliferation	Breast cancer, prostate cancer, lung cancer, renal cancer	Cytoplasm	[98,99,102,103]
snoRNA	60–300	It is generally encoded by the introns or protein coding sequences of the DNA, but there it can also be encoded by DNA regions, where the snoRNA host genes (SNHGs) are located	RNA POL II or RNA POL III	snoRNAs in the intronic region show high degree of conservation	rRNA processing (2′-O-methylation or pseudouridylation, pre-rRNA cleavage) (H/ACA box snoRNA); mRNA splicing and mRNA processing (C/D box snoRNA), small Cajal body RNA (scaRNA), snoRNAs are origin for miRNAs	Activated in oxidative stress; involved in glucose metabolism; cell death, proliferation, invasion and metastasis	Lung cancer, prostate cancer, breast cancer, acute myeloid leukemia, acute promyelocytic leukemia, colorectal cancer, hepatocellular carcinoma, glioblastoma, osteosarcoma, pancreatic cancer	Nucleus (nucleolus), cytoplasm (only during cellular stress)	[20,104,105,106]
snRNA	100–300	Encoded by 5 DNA regions: U1, U2, U4, U5, and U6	RNA POL III (U6), RNA POL II (U1-U5)	High degree of conservation	pre-mRNA splicing	Cell cycle progression, Tumorigenesis, oncogenic development	Breast cancer, lung cancer	Nucleus (main function), can also be exported into the cytoplasm (U6)	[107,108,109,110,111,112]
vtRNA	88–100	Special sequence from the DNA, vault DNA sequences are located on chromosome 5	RNA POL III	High degree of conservation	Associated with specific proteins vault proteins in the cytoplasm, forming vault RNPs in the cytoplasm	Autophagy, intracellular and membrane trafficking, multidrug resistance (drug export from the cytoplasm)	Breast cancer, lymphoma, lung cancer, multiple myeloma	Cytoplasm	[22,113,114,115,116]
Alu-element RNA	around 250	Alu-repeat containing transposable elements, comprises around 10% of human genome	RNA POL II, RNA POL III	The main genes containing ALU repeats are conserved, but there are also a number of evolved pseudogenes through single base substitution	They are involved in protein translation, are the ancestors of epigenetic enhancers, they offer new binding sites for transcription factors, impair mRNA or miRNA transcription	Increased expression in stress conditions, involved in the epithelial-to-mesenchymal transition, cell cycle progression	Breast cancer, colorectal cancer	Nucleus	[23,24,25,26]
piRNA	21–35	Encoded by protein-coding genes (untranslated regions of messenger RNAs), sequences form intergenic regions (long intergenic regions)	RNA POL III	High degree of conservation	It has a close interaction with piwi protein and together are involved in RNA cleavage. It also has epigenetic functions through heterochromatin regulation and induced changes in DNA methylation pattern.	Protects against germline genome stability and DNA integrity; maintains cancer stemness, apoptosis impairment, involved in telomerase activity, cell cycle progression and metastasis	Gastric cancer, lung cancer, cervical cancer, hepatocellular cancer, breast cancer, colorectal cancer, ovarian cancer	Cytoplasm	[117,118,119,120]
